# Statistic and Network Features of RGB and Hyperspectral Imaging for Determination of Black Root Mold Infection in Apples

**DOI:** 10.3390/foods12081608

**Published:** 2023-04-10

**Authors:** Wen Sha, Kang Hu, Shizhuang Weng

**Affiliations:** 1 School of Electrical Engineering and Automation, Anhui University, 111 Jiulong Road Hefei, Hefei 230601, China; 2 Engineering Research Center of Autonomous Unmanned System Technology, Ministry of Education, Anhui University, 111 Jiulong Road Hefei, Hefei 230601, China; 3 National Engineering Research Center for Agro-Ecological Big Data Analysis & Application, Anhui University, 111 Jiulong Road Hefei, Hefei 230601, China

**Keywords:** apple, fungal infection, hyperspectral imaging, RGB imaging, deep learning

## Abstract

Apples damaged by black root mold (BRM) lose moisture, vitamins, and minerals as well as carry dangerous toxins. Determination of the infection degree can allow for customized use of apples, reduce financial losses, and ensure food safety. In this study, red-green-blue (RGB) imaging and hyperspectral imaging (HSI) are combined to detect the infection degree of BRM in apple fruits. First, RGB and HSI images of healthy, mildly, moderately, and severely infected fruits are measured, and those with effective wavelengths (EWs) are screened from HSI by random frog. Second, the statistic and network features of images are extracted by using color moment and convolutional neural network. Meanwhile, random forest (RF), K-nearest neighbor, and support vector machine are used to construct classification models with the above two features of RGB and HSI images of EWs. Optimal results with the 100% accuracy of training set and 96% accuracy of prediction set are obtained by RF with the statistic and network features of the two images, outperforming the other cases. The proposed method furnishes an accurate and effective solution for determining the BRM infection degree in apples.

## 1. Introduction

Rich in nutrition with many vitamins and minerals, apples are one of the most widely grown fruits in the world [1]. However, in the process of its growth, harvest, transportation, and sale, apple fruits are vulnerable to infection of fungal diseases [2]. Among these diseases, the black root mold (BRM) is common and severe. BRM can cause fruit rot and produce harmful metabolites to affect quality and increase food safety risks, thereby inducing economic and trade losses. Different treatments are available for apple fruits with different infection degrees. Apples without infections are considered high-quality fruits, and those with fungal infections are used as processed products, livestock feed, or plant fertilizer. Traditional diagnosis methods of fungal infection degree in fruits—such as manual inspection and liquid chromatography—are subjective, time-consuming, or complex. Therefore, developing a rapid and accurate detection method of fungal infection degree in apple fruit is an urgent matter.

With the rise of smart agriculture, spectroscopy and imaging techniques—including near infrared spectroscopy (NIRS), red-green-blue (RGB) imaging, and hyperspectral imaging (HSI)—have become important detection means for the infection of fungal diseases in plants [3]. For example, the NIRS method was proposed for classifying heathy and fusarium head blight-infected wheat kernels [4]. However, NIRS can obtain only the point features and cannot realize massive and rapid classification. RGB imaging can obtain the shape, color, and texture with low cost and rapid speed, and has been used for early detection of diseases in apples [5] and orchids [6]. However, the highly correlated channels in RGB images cannot provide the comprehensive information of targets, thereby limiting the detection accuracy. HSI integrates digital image and spectroscopy to visualize the spatial distribution of appearance features, internal composition, and structure [7]. A real-time pixel-based early apple bruise detection system based on HSI was developed [8]. Nonetheless, the spatial resolution of HSI images is generally low to perceive refined external characteristics. By contrast, RGB images show high spatial resolutions. RGB and HSI fusions have been used for plant disease detection. HSI and high-resolution RGB images are also fused for detecting potential diseases in banana leaves [9]. Therefore, HSI and RGB are combined to explore the detection of fungal infection in apple fruits.

Representations of color, shape, texture, and spatial relationships in images can be obtained by statistic methods on features such as histogram, color moments, and grey-level co-occurrence matrix. Statistic methods treat the image in terms of a matrix with pixel values as elements and are supported by a rigorous theory and express related image information in a simple and intuitive manner. In the work of Zhang et al., statistic features are obtained by extracting the color, texture, and shape of the images through the grey-level co-occurrence matrix to identify the extent of Fusarium damage in wheat kernels [10]. However, statistic features are ineffective in capturing hidden complex non-linear information and responding to sudden changes in data behavior. Network features are the abstract information representation obtained from automatic feature extraction of images by deep learning networks, such as VGGNet, AlexNet, Inception, and convolutional neural networks (CNN). Network features reflect the deep mining of image information and effective extraction of discriminative information. These methods are widely used in the field of pattern recognition, including the recognition of plant disease [11] and fruit quality [2], with their strong generalization of features as well as robustness to background, illumination, occlusion, and noise. Network features extracted by CNN are also to detect damages to apple surfaces and automatically classify different fungi diseases [2]. Nevertheless, network features lack theoretical explanation and their quality is susceptible to training and structure of learning networks, and thus they are extremely unreliable for small samples [12]. Statistic and network features describe the image information with respective advantages, and thus multi-feature fusion has become an effective method to enhance detection. For example, a model that fuses statistic features extracted by a histogram of oriented gradient and network features extracted by CNN effectively captures the local spatial texture information in plant leaf images [13].

Furthermore, given that the HSI images are hundreds dimension and contain a large amount of redundant information, direct treatment causes high computational complexity and time costs. Screening the images of effective wavelengths (EWs) from HSI has been determined as a feasible measure [14], because the monochrome images of the several specific wavelengths intrinsically provide abundant spectroscopic and image information [10].

**Figure 1:**
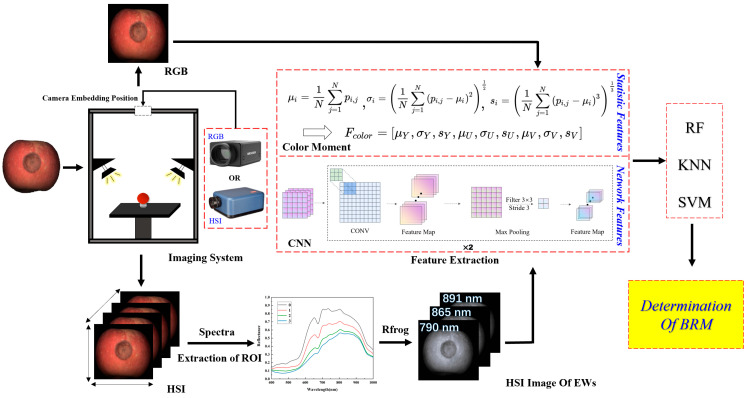
Flow chart of detection of BRM infection degree in apples. In the color moment, *u_i_*, σ*_i_* and *s_i_* represent the first-order moments, second-order moments and third-order moments of the image color, *p_ij_* represents the i-th color component of the j-th pixel of image, N represents the number of pixels in the image, and *F_color_* represents the histogram vectors including the first three orders of color moment for the three components (Y, U, V) of image, where Y denotes luminance, U and V denote chrominance.

In this study, the multi-features of RGB and HSI are combined to identify the infection degree of BRM in apple fruits (Figure 1). First, RGB and HSI images of apples with different fungal infection degrees are measured, and the images of EWs are extracted based on RFrog from HSI images. Then, the statistic and network features of RGB and HSI images of EWs are extracted by using color moment and CNN. Finally, random forest (RF), K-nearest neighbor (KNN), and support vector machine (SVM) are used to construct classification models based on the various features.

## 2. Materials and Methods

### 2.1. Samples

**Figure 2:**
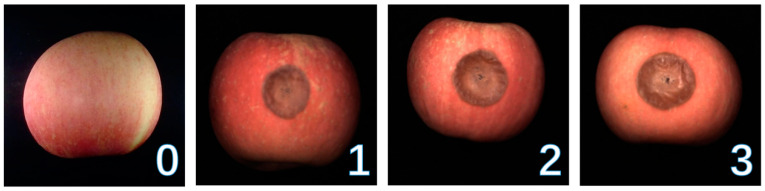
Images of apples with different degrees of infection. 0: healthy, 1: mildly infected, 2: moderately infected, 3: severely infected.

Apple fruits were purchased from the alpine orchards in Aishan, Yantai City, on four occasions to increase the sample diversity. Apples with consistent ripeness, uniform fruit shape, smooth appearance, red color, and no damage condition were selected for analysis. A total of 230 apple samples were prepared, of which 178 were inoculated with Rhizopus Stolonifer (RS). The inoculated samples were stored in a storage cabinet at 25 °C and 99% relative humidity for four days. RS-infected apple samples showed different degrees with the change in external information and internal components of decay over time, and the infection degree was labelled according to the time of infection (Figure 2). The infection degree was mainly proportional to the elapsed time since the infection. The rotted area became larger and colorful, and the rotted depth deepened as the time was elapsed. Samples at 2, 3, and 5 days after infection were acquired and regarded as mildly infected, moderately infected, and severely infected. Among the 178 infected apples, 62 were mildly infected, 61 were moderately infected, and 55 were severely infected. The remaining 52 apples were used as the healthy sample (Table S1).

### 2.2. Image Acquisition

A digital imaging instrument (MV-CE060-10UC, Hikvision, Hangzhou, China) was used to obtain the RGB images of apples. The sample must be completely within the required field of view. Based on the apple samples of 80–90 mm and field of view of 450 mm × 300 mm, the distance of the sample from the lens was set to 500 mm. The focal length of the lens was calculated as shown in Equation (1).

(1) $$f=\frac{WD\times V}{H}=\frac{500\times 7.38}{450}=8.2$$

where *f* is the focal length of the lens to be obtained, *WD* is the distance from the lens to the object, *V* is the width of the camera target surface, and *H* is the width of the captured field of view. According to Equation (1), the camera was equipped with a Hikvision optical lens (MVL-HF0828M-6MPE) with six megapixels and a focal length of 8 mm.

The HSI images of apples were measured by using the visible/NIR HSI system containing a hyperspectral imager (SOC710VP, Surface Optics Corporation, San Diego, CA, USA), a dark box, and a computer. The spectral response range of the hyperspectral imager covered 260 wavelengths from 400 nm to 1000 nm, and the range was also the commonly used interval for sensing infection. The dark box included a carrier table, which could be moved up and down to place samples, and two tungsten halogen lamps (150 W). The computer was assembled with Hyper Scanner software for setting parameters in the image acquisition, such as resolution and exposure time.

Apples were placed on a black plate, and the plate was placed on the carrier table. To ensure that only apple samples were in the captured HSI image, we set the distance between the carrier table and the lens to 50 cm.

To reduce dark frame noise in any electronic imaging system, researchers use the black and white correction, as shown in Equation (2):

(2) $$R=\frac{R_{0}-B}{W-B}\times 100\%$$

where *R*_0_ is the raw image, *W* is the standard whiteboard image, *B* denotes the dark reference image, and *R* refers to the corrected spectral image.

### 2.3. Reflectance Spectra and Images of EWs from HSI

For the acquired HSI images, the apples were separated from the background by threshold segmentation to obtain the grayscale images, which were then transformed into binary images and masked to obtain the ROI images. The spectra of the sample from the ROI region were averaged as the reflectance spectra of the apple. The EWs of spectra were filtered by using RFrog. Derived from reversible jump Markov chain Monte Carlo, RFrog generates a random subset of initial variables and then iteratively generates a subset of candidate variables based on the regression coefficient. After reaching the iteration number, the probability of each selected variable is calculated [15]. In RFrog, the selection probability of each wavelength is calculated to evaluate the importance, and three EWs of 790, 865, and 891 nm with the first three importance of 0.986, 0.983, and 0.977 were screened. The monochrome images of the three wavelengths were extracted from HSI images and superposed as EW images.

### 2.4. Feature Extraction of Images

The commonly used method for color feature extraction is color moments. This study also used color moments to obtain colors as statistic features of samples with different infection degrees. The color distribution information was mainly concentrated in the lower order moments, where the first (mean), second (variance), and third (skewness) represent three color features of overall lightness and darkness, color distribution range, and symmetry, respectively [16]. Finally, nine color features were obtained for each sample.

CNN can construct information by fusing spatial and channel-wise features within the local field of perception of each layer and capture images by combing local information at the high level [17]. These traits provide the massive potential of CNN in image feature extraction. The obtained RGB and EW images were cropped to a fixed size and separately imported into the CNN to extract the network features. The CNN consisted of two convolutional layers with 32 and 64 3 × 3 kernels. The size of the pooling layer was also set to 3 × 3. The features of the two convolutional layers were concentrated as network features.

### 2.5. Classification Methods

By using RF, KNN, and SVM, classification models based on multiple image features were developed to identify the infection degree of BRM in apples. RF is a combination of tree predictors. With slight modifications to bagging, the method requires only a small amount of tuning parameters and can naturally rank the importance of features to run efficiently on large datasets and obtain accurate classification performance. A selection of regression random variables was used for sampling, generating a decision tree, and forming a forest [18]. KNN is a relatively simple and effective method. For classifying the test sample, KNN finds the known *k* samples that are most similar. Then, the classification of test samples is determined based on the categories of the *k* samples [19]. SVM is a statistic learning method based on risk minimization theory. By using the kernel function, the data of different classes are separated by a hyperplane, which is maximized by optimizing the support vectors [20].

### 2.6. Performance Evaluation

To better evaluate the discriminative ability of models, we divided the 230 collected samples into training and prediction sets in a ratio of 2:1. The classification model was built by using the 154 samples from the training set, and its performance was tested by using the 76 samples from the prediction set. Precision (*P*), recall (*R*), F1-score (*F*1), and accuracy (*ACC*) were used for the quantitative evaluation of the models. Their calculations are shown below. The receiver operator characteristic curve (ROC) and the confusion matrix reflected the model performance. The color moments and CNN used for extracting image features and the RF, KNN, and SVM classification models were based on Python programming.

(3) $$P=\frac{TP}{TP+FP},R=\frac{TP}{TP+FN},F1=\frac{2P\times R}{P+R},ACC=\frac{TP+TN}{TP+FN+FP++TN}$$

where *TP* (true positive) represents the number of positive samples identified as positive samples, *FP* (false positive) represents the number of negative samples identified as positive samples, *FN* (false negative) represents the number of positive samples identified as negative samples, and *TN* is true negatives, which represents the number of negative samples identified as negative samples.

## 3. Results and Discussion

### 3.1. Detection of BRM Infection Degrees of Apples Using RGB

Statistic and network features extracted from RGB using color moments and CNN were combined with RF, KNN, and SVM to develop the classification models of BRM infection in apples (Table 1). KNN achieved the highest fitting results but the lowest prediction results. ACC_T_ of 100% and ACC_P_ of 86.8% and 87.5% were obtained for statistic and network features. This is mainly because KNN complicates the distance calculation for each dimension leading to the occurrence of model overfitting when data have high dimensionality. In addition, for statistic features, RF and SVM showed better classification with ACC_P_ of 90.7% given its excellent ability of complex nonlinear modeling. In addition, RF achieved the high fitting results with the ACC_T_ of 100%. However, recall showed that RF had a misclassification of moderate infection to mild infection. In terms of network features, the best classification results were still obtained by RF with ACC_P_ of 95.1%, with an improvement of approximately 4% compared with statistic features.

Notably, statistic and network features exhibited respective advantages in categorizing various infection degrees. In terms of severe degree, the F1-score of statistic features was higher than network features on both RF, KNN, and SVM models, indicating that the statistic features of RGB have the advantage of distinguishing severity. Network features with the optimal classification model, RF, obtained a recall of 81.8% for the moderate degree, which was better than the statistic features with recall = 68.7%. In general, the statistic and network features of RGB can effectively classify the BRM infection of apples, but the accuracy is insufficient for practical applications.

### 3.2. Detection of BRM Infection Degree of Apples Using HSI Images of EWs

**Figure 3:**
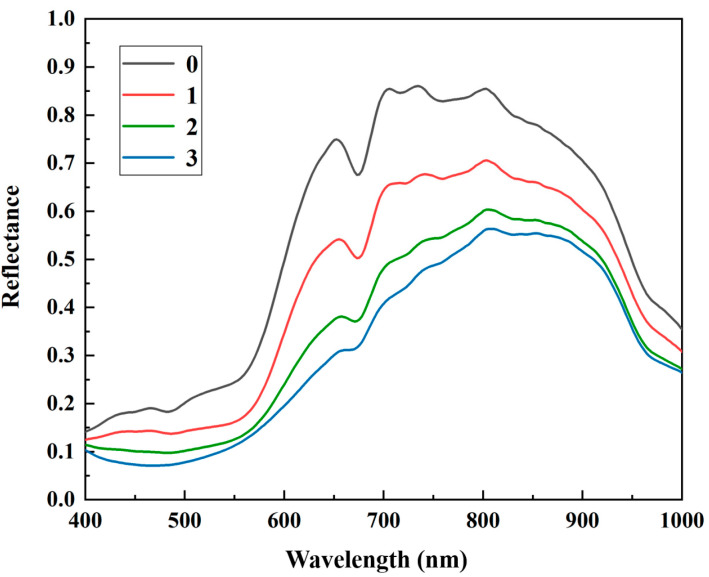
Spectra of apples with different infection degrees; 0: healthy, 1: mildly infected, 2: moderately infected, 3: severely infected.

The average spectra extracted from HSI images of various infection degrees of BRM in apple samples (Figure 3) showed a similar reflectance trend with an increase to approximately 850 nm and then decrease. An apparent reflectance rise appeared in the range of 500–650 nm, and a chlorophyll-induced valley occurred at 650–680 nm. The band at 700–740 nm can be assigned to the oxyhydrogen (O-H) extension and the third and fourth overtones of hydrocarbon (C-H) extension in sugar. The band of 960 nm was attributed to O-H and the second-order overtone of water [21]. As the infection degree increased, the spectral reflectance and intensity of characteristic peak gradually decreased because of physical characteristics and chemical composition changes, such as tissue color changes, water loss, sugar content reduction, and organic acid oxidation. These phenomena preliminarily demonstrated the detection feasibility of BRM infection degree using HSI images of EWs carrying the above critical information.

The EW images with 790, 865, and 891 nm were screened based on the importance of RFrog, and the color moments and CNN were used to extract their statistic and network features. These two features were combined to build the classification models of BRM infection degree in apples by using RF, KNN, and SVM (Table 2). The use of HSI images of EWs improved the overfitting phenomenon of KNN in experiment 3.1 for both statistic or network features with the result of ACC_T_ = 100% and above 90% ACC_P_. For statistic features, RF, KNN, and SVM obtained the classification with ACC_P_ of 92.1%, 90.7%, and 93.4%, respectively, which were both higher than the ACC_P_ from RGB of 90.7%, 86.8%, and 90.7%, respectively. For network features, the overall better results were obtained with ACC_P_ of 93.4%, 92.1%, and 94.7% for RF, KNN, and SVM, respectively. The optimal classification was obtained by using SVM.

Overall, the detection of BRM infection degree of apples using HSI images of EWs improved the predictive ability, although results were better using RGB on certain classification tasks. For example, the RF from the network of RGB obtained an ACC_P_ of 95.1%, a higher result than HSI. Given the advantages of RGB and HSI images of EWs in classifying the BRM infection degrees in apples, attempts to combine their features are worthwhile.

### 3.3. Identification of BRM Infection Degree by Multi-Features

The statistic and network features of RGB and HSI images of EWs were fused to develop the classification models of BRM infection degree by using RF, KNN, and SVM (Table 3). Given the unavoidable redundancy of statistic features, Pearson’s correlation coefficient was used for screening (Figure S1). Specifically, one of the statistic features with correlation coefficients higher than 0.4 was as an alternative to remove. If the feature had a low correlation with other features, it was still retained. On the contrary, the feature was removed. Nine features were excluded from the eighteen color features. As for network features, 1 * 1 convolution at the end of the network set the number of output channels to half of the original one to ensure that the number of features remained consistent for multi-features fusing.

The fusion of multi-features of two images resulted in better detection. In terms of model fitting ability, the ACC_T_ of 92.8% and 98.7% were obtained by SVM for the statistic and network features of RGB, and the ACC_T_ of 99.3% was acquired by RF for the statistic features of HSI images of EWs. However, the RF and SVM all obtained ACC_T_ of 100% due to the fusion of multi-features of two images. The possible reason was that the various features enhanced the information distribution to increase the fitting ability of the models. The high value of ACC_T_ showed good fit for all three classification models. However, the ACC_P_ remained at above 90%, which indicated no obvious overfitting occurred. The ACC_P_ of RF, KNN, and SVM also increased to 98.6%, 98.6%, and 96.0%. Among them, the overfitting of KNN gained considerable improvement by comparison with the use of single-type features from the RGB or HSI images of EWs. KNN showed misclassifications of healthy and moderately infected apples, which is a serious fault in practical application. By comparison, RF achieved better results as its misclassification were between mildly and moderately infected samples. Specifically, the precision clearly increased for the detection of mildly infected apples from 79.1% to 95%, recall of moderately infected ones from 68.7% to 95.4%, and F1-score from 81.4% to 97.6% in RF. These results may indicate that multi-features were cancellable for the misclassification of moderately infected apples to a great extent.

**Figure 4:**
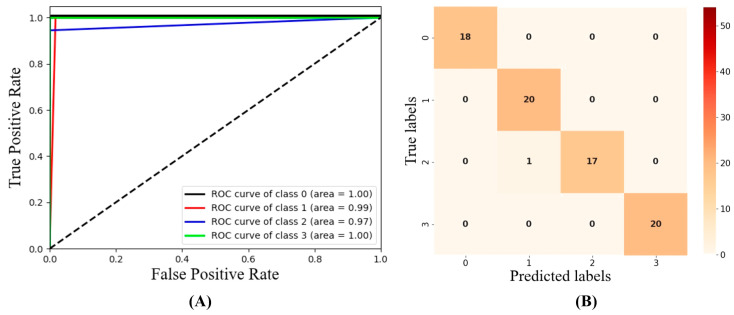
ROC curves (**A**) and confusion matrix (**B**) for optimal results with RF based on RGB and HSI images of EWs’ fusion features.

Meanwhile, the ROC curve and AUC were adopted to evaluate RF (Figure 4A). As the ROC curve approached the upper left part and the AUC value approached 1, the power and performance of classifiers increased. As shown in the figure, the ROC curve of RF was concentrated on the upper left, and the AUC values were all over 0.9 and even reached 1.0 on the healthy and severely infected apples. In addition, the confusion matrix of RF showed that only one moderately infected apple was labelled as mildly infected (Figure 4B). Thus, RF combined with the fused features from RGB and HSI images of EWs obtained the accurate determination of BRM infection degree in apples.

In recent years, numerous researchers have explored the quality determination of fruits by using RGB and HSI. By using RGB, fruit quality is generally evaluated based on visual appearance, such as color, texture, and shape. For instance, the color features of mango were extracted from RGB to determine mango disease [22]. However, the R, G, and B channels in RGB images cannot effectively determine the internal characteristics of fruits, which limits the detection accuracy. Recently, possessing the spatial distribution of appearance characteristics, internal composition, and structure, HSI has been adopted for the detection of external damage and internal components in fruits. Zhu and Li used HSI to identify the bruised apples in five stages (1 min, 1 day, 2 days, 3 days, and 4 days after bruising) with the overall classification accuracy of 92.9% [23]. Weng et al. detected the soluble solid content, pH, and vitamin C of strawberry by using the spectral and color features extracted from HSI [24]. However, HSI with low spatial resolution cannot perceive the refined external characteristics of fruits. This problem can be alleviated by integrating RGB with high-resolution external information and HSI with good internal composition. In practical application, screening the images of EWs from HSI is a feasible approach to avoid excessive computation and complexity and ensure abundant information.

Meanwhile, statistic features are generally used to represent the image information for both RGB and HSI. The statistic features obtained by histogram, color moment, or gray co-occurrence matrix are adopted to describe the color, shape, texture, and spatial relationship in images. Considering the apparent change in color of the BRM-infected apples, the color distributions are extracted by color moments [25] as statistic features in this study. Instead of manually designed features, the network features extracted by deep networks can capture the hidden complex nonlinear information and the high-level semantic information in images. Network features have been proven to be of great value in the inspection of surface defects in apples [2] and the measurement of sugar content in strawberries [26]. The multi-features commonly analyze the quality of fruits because multi-features describe multiple sources of information for the analysis of fruits. Hlaing and Zaw combined texture and color features for the classification of tomato plant diseases [27]. Given statistic and network features detection of the multi-level characteristics of images, the fusion of the two features is expected to improve the infection analysis.

Based on this, the statistic and network features from RGB and HSI images of EWs were used to determine the BRM infection degree in apples. The use of RGB with external information and HSI images of EWs with internal composition allows for rich and comprehensive descriptions of fungus infection. The utilization of multi-features further strengthens the advantage. High-quality detection of BRM infection degree with the ACC_P_ = 98.6% are obtained, outperforming the single feature detection of a single image with ACC_P_ = 90.7%, 95.1%, 93.4%, and 94.7%.

## 4. Conclusions

In this study, the statistic and network features of RGB and HSI images of EWs are combined to detect the BRM infection degree in apples. First, the individual features of two images are used and then their multi-features are combined to determine the BRM infection degree. RF achieved the best results with ACC_T_ of 100% and ACC_P_ of 98.6%, outperforming cases of individual features. Moreover, the average AUC of 0.98 indicated that models with multi-features obtain results with excellent robustness. In summary, the proposed method provides a feasible scheme for determining the BRM infection degree in apples and presents wide application prospects in fruit quality. In future, infections of various fungi and more fruit species can be explored to generalize the application of the proposed method. Novel and powerful feature extraction and modeling methods will also be attempted to enhance the characteristic description and recognition performance. Developing simple and low-cost equipment to obtain RGB images and images of sporadic and specific wavelengths in one stage builds a reliable and customized support for fruit infection detection.

## Figures and Tables

**Table 1:** Classification results of BRM infection degree in apples using RF, KNN, and SVM with RGB.

Features	Methods	Classes	ACC (%)	P (%)	R (%)	F1 (%)
Statistic	RF	Healthy	ACC_T_ = 100 ACC_P_ = 90.7	100	100	100
		Mildly infected		79.1	100	88.3
		Moderately infected		100	68.7	81.4
		Severely infected		100	100	100
	KNN	Healthy	ACC_T_ = 100 ACC_P_ = 86.8	100	70.0	82.3
		Mildly infected		90.4	100	95.0
		Moderately infected		68.1	93.7	78.9
		Severely infected		94.7	85.7	90.0
	SVM	Healthy	ACC_T_ = 92.8 ACC_P_ = 90.7	86.9	86.9	86.9
		Mildly infected		100	94.7	97.2
		Moderately infected		90.4	86.3	88.3
		Severely infected		85.7	100	92.3
Network	RF	Healthy	ACC_T_ = 100 ACC_P_ = 95.1	92.0	100	95.8
		Mildly infected		100	100	100
		Moderately infected		100	81.8	90.0
		Severely infected		85.7	100	92.3
	KNN	Healthy	ACC_T_ = 100 ACC_P_ = 87.5	82.6	95.0	88.3
		Mildly infected		88.8	84.2	86.4
		Moderately infected		90.9	62.5	74.0
		Severely infected		83.3	95.2	88.8
	SVM	Healthy	ACC_T_ = 98.7 ACC_P_ = 87.7	88.4	100	93.8
		Mildly infected		100	68.4	81.2
		Moderately infected		86.9	90.9	88.8
		Severely infected		85.7	100	82.3

**Table 2:** Classification results of BRM infection degree in apples using RF, KNN, and SVM with HSI images of EWs.

Features	Methods	Classes	ACC (%)	P (%)	R (%)	F1 (%)
Statistic	RF	Healthy	ACC_T_ = 99.3 ACC_P_ = 92.1	100	95.0	97.4
		Mildly infected		86.3	100	92.6
		Moderately infected		82.3	87.5	84.8
		Severely infected		100	85.7	92.3
	KNN	Healthy	ACC_T_ = 100 ACC_P_ = 90.7	90.0	90.0	90.0
		Mildly infected		88.2	78.9	83.3
		Moderately infected		84.2	100	91.4
		Severely infected		100	95.2	97.5
	SVM	Healthy	ACC_T_ = 100 ACC_P_ = 93.4	100	90.0	94.7
		Mildly infected		82.0	100	90.4
		Moderately infected		93.7	93.7	93.7
		Severely infected		100	90.4	95.0
Network	RF	Healthy	ACC_T_ = 100 ACC_P_ = 93.4	95.5	100	100
		Mildly infected		79.1	100	88.3
		Moderately infected		100	88.7	81.4
		Severely infected		100	100	100
	KNN	Healthy	ACC_T_ = 100 ACC_P_ = 92.1	100	95.0	97.4
		Mildly infected		85.7	94.7	90.0
		Moderately infected		81.2	81.2	81.2
		Severely infected		100	95.2	97.5
	SVM	Healthy	ACC_T_ = 100 ACC_P_ = 94.7	100	100	100
		Mildly infected		82.6	100	90.4
		Moderately infected		100	75.0	85.7
		Severely infected		100	100	100

**Table 3:** Classification results of BRM infection degree in apples using RF, KNN, and SVM with fused features.

Fusion	Methods	Classes	ACC (%)	P (%)	R (%)	F1 (%)
Features from RGB and HSI	RF	Healthy	ACC_T_ = 100 ACC_P_ = 98.6	100	100	100
		Mildly infected		95.0	100	97.4
		Moderately infected		100	95.4	97.6
		Severely infected		100	100	100
	KNN	Healthy	ACC_T_ = 100 ACC_P_ = 98.6	100	95.0	97.4
		Mildly infected		100	100	100
		Moderately infected		94.1	100	96.9
		Severely infected		100	100	100
	SVM	Healthy	ACC_T_ = 100 ACC_P_ = 96.0	100	100	100
		Mildly infected		86.3	100	92.6
		Moderately infected		100	81.2	89.6
		Severely infected		100	100	100

## Data Availability

The data presented in this study are available on request from the corresponding author.

## Supplementary Materials

The following supporting information can be downloaded at: https://www.mdpi.com/article/10.3390/foods12081608/s1, Figure S1: Pearson correlation analysis of 18 color features. Table S1: RGB and HSI Imaging system to capture apple data.
