# Supplementary material for: Statistic and Network Features of RGB and Hyperspectral Imaging for Determination of Black Root Mold Infection in Apples
Source: Foods. 2023 Apr 10;12(8):1608. doi: 10.3390/foods12081608 (PMC10137991; doi:10.3390/foods12081608)
Supplement: Supplementary file 1 [file foods-12-01608-s001.zip › foods-2263102-supplementary.pdf]

Supplementary Material

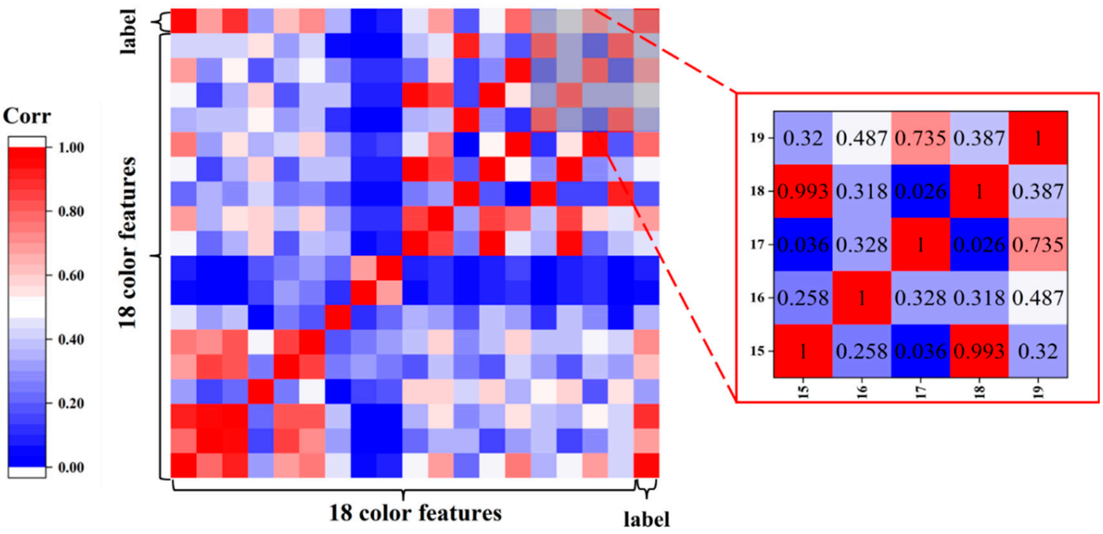

Figure S1. Pearson correlation analysis of 18 color features.

Table S1. RGB and HIS Imaging system to capture apple data.

Degree	Heathy	Mildly infected	Moderately infected	Severely infected
Number (RGB)	52	62	61	55
Number (HSI)	52	62	61	55
